# The external PASTA domain of the essential serine/threonine protein kinase PknB regulates mycobacterial growth

**DOI:** 10.1098/rsob.150025

**Published:** 2015-07-01

**Authors:** Obolbek Turapov, Jessica Loraine, Christopher H. Jenkins, Philippe Barthe, Daniel McFeely, Francesca Forti, Daniela Ghisotti, Dusan Hesek, Mijoon Lee, Andrew R. Bottrill, Waldemar Vollmer, Shahriar Mobashery, Martin Cohen-Gonsaud, Galina V. Mukamolova

**Affiliations:** 1 Department of Infection, Immunity and Inflammation, University of Leicester, Leicester LE1 9HN, UK; 2 Centre de Biochimie Structurale, CNRS UMR 5048, 29, rue de Navacelles, Montpellier 34090, France; 3 INSERM U1054, Université Montpellier I et II, Montpellier, France; 4 Dipartimento di BioScienze, Università degli Studi di Milano, Milan, Italy; 5 Department of Chemistry and Biochemistry, 423 Nieuwland Science Center, University of Notre Dame, Notre Dame, IN 46556, USA; 6 Centre for Bacterial Cell Biology, Institute for Cell and Molecular Biosciences, Newcastle University, Newcastle upon Tyne NE2 4AX, UK

**Keywords:** *Mycobacterium tuberculosis*, serine/threonine protein kinase, magnesium, muropeptides, PknB, PASTA domain

## Abstract

PknB is an essential serine/threonine protein kinase required for mycobacterial cell division and cell-wall biosynthesis. Here we demonstrate that overexpression of the external PknB_PASTA domain in mycobacteria results in delayed regrowth, accumulation of elongated bacteria and increased sensitivity to β-lactam antibiotics. These changes are accompanied by altered production of certain enzymes involved in cell-wall biosynthesis as revealed by proteomics studies. The growth inhibition caused by overexpression of the PknB_PASTA domain is completely abolished by enhanced concentration of magnesium ions, but not muropeptides. Finally, we show that the addition of recombinant PASTA domain could prevent regrowth of *Mycobacterium tuberculosis*, and therefore offers an alternative opportunity to control replication of this pathogen. These results suggest that the PknB_PASTA domain is involved in regulation of peptidoglycan biosynthesis and maintenance of cell-wall architecture.

## 1. Introduction

Serine/threonine protein kinases (STPKs) are widely distributed in Gram-positive and Gram-negative bacteria [1]. *Mycobacterium tuberculosis*, the causative agent of tuberculosis, possesses 11 STPKs [2] and two of them, PknA and PknB, are indispensable for growth in laboratory culture [3–5], while PknE [6], PknG [7,8] and PknH [9] have been implicated in *M. tuberculosis* virulence. The essential PknB kinase belongs to a distinct family of STPKs found only in Gram-positive bacteria [10]. The important feature of these kinases is the presence of the so-called PASTA (penicillin-binding protein and serine/threonine kinase associated) domains in the surface-exposed region [11]. In Firmicutes, PASTA-domain-containing kinases are not essential for growth. In *Staphylococcus aureus*, an Stk1 mutant was impaired in virulence and had higher resistance to Triton X-100 and fosmidomycin [12], while in *S. pneumoniae* StkP kinase was important for competence, biofilm formation and virulence [13]. More detailed investigation of the role of StkP in *S. pneumoniae* established that it has a crucial role in coordinating cell division and peptidoglycan synthesis during growth [14]. By contrast, PrkC in *Bacillus subtilis* was shown to be important for survival in stationary phase. A *prkC* deletion mutant had a significantly lower optical density in stationary phase compared with the wild-type bacilli [15]. However, in later studies it was demonstrated that PrkC regulated a novel muropeptide-mediated germination pathway [16] and possibly remodelling of the cell wall via controlling expression of YocH muralytic enzyme [17].

In *Streptomyces coelicolor*, a PASTA domain containing kinase PknB regulates carbon flux and antibiotic production, and is not essential for growth [18]. In *Corynebacterium glutamicum*, PknB is also dispensable for growth, and a significant change in replication and cell shape could only be detected in mutants missing several STPKs [19]. In this bacterium, PknB apparently regulates polymerization of FtsZ; however, the precise mechanism and biological significance of this observation require further investigation.

Mycobacteria appear to be a unique bacterial group in which PknB is essential for growth [3–5]. Its overexpression or partial depletion in *M. smegmatis* and *M. bovis* BCG caused dramatic alterations of cellular morphology and growth inhibition; the enzymatic activity of PknB was demonstrated to be essential for the observed effects [4,5]. Over the past decade, great progress has been achieved in the identification of PknB substrates [10]. They include proteins from various functional categories: cell-wall enzymes—InhA [20], PbpA [21] and MabA [22]; regulatory proteins—SigH [23], GarA [24] and FhaA [25]; and proteins involved in cell division—Wag31 [4] and MviN [26]. Also, PknB regulates an ‘oxygen-mediated replication switch’, and hence transition to dormancy and resuscitation [27]. Therefore, it is not surprising that PknB has been considered as a critical drug target for development of anti-tuberculosis antimicrobials. Possible application of PknB kinase inhibitors for killing of *M. tuberculosis* was investigated. Yet only limited success has been achieved, mainly because of poor penetration of these agents into mycobacterial cells [28].

**Figure 1.**
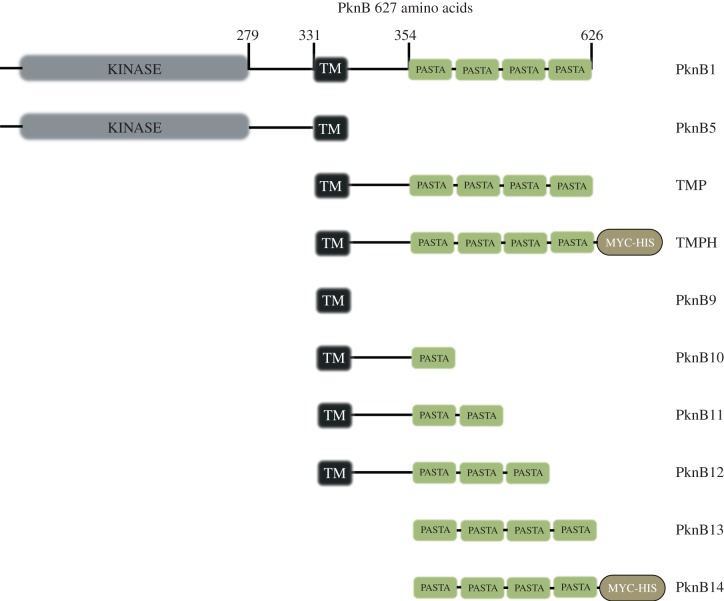
Schematic representation of PknB constructs and strains generated in this study. Kinase, N-terminal kinase domain (aa 1–279); TM, transmembrane region (aa 331–354); TM-PASTA, penicillin and serine or threonine kinase-associated domain (aa 331–627); MYC-HIS, His-Tag; PASTA (aa 354–627).

PknB consists of several domains (figure 1); functions for some of them have been established, while the precise role of others remains unknown [29]. The components of PknB include a conserved catalytic kinase domain, a juxtamembrane part attached to a membrane-spanning region and surface-exposed sensory component, consisting of PASTA, designated as PknB_PASTA domain [30,31]. PASTA domains have been proposed to recognize growing strands of nascent peptidoglycan and activate STPKs. Structural studies showed that *B. subtilis* PASTA domain binds synthetic muropeptides at relatively high affinity, with the presence of the diaminopimelic acid in the muropeptide being crucial for this binding [32]. The PknB_PASTA domain from mycobacteria can also bind synthetic muropeptides; however, it remains unclear whether this binding influences activation of PknB, bacterial growth and resuscitation [33].

In this study, we investigated whether the extracellular PknB_PASTA domain itself plays a distinct role in mycobacterial growth, and therefore can be potentially used as a drug target or a novel chemotherapeutic agent. The results presented here suggest that the PASTA domain probably recognizes growing peptidoglycan strands, and regulates production of penicillin-binding proteins and distribution of proteins involved in division and generation of septum. Furthermore, our data indicate that the interactions of the PknB_PASTA domain with the cell wall are important, and that its abundance may serve as a controlling mechanism for the integrity of the cell wall and bacterial growth.

## 2. Material and methods

### 2.1. Bacterial strains and growth media

*Mycobacterium smegmatis* mc2155, *M. bovis* BCG Glaxo strain and *M. tuberculosis* H37Rv were grown in Sauton's medium or Middlebrook 7H9 (Becton, Dickinson and Company) medium supplemented with Albumin-Dextrose Complex (designated as supplemented 7H9 medium). *M. smegmatis* was also grown in Lysogeny broth (LB) with addition of 0.05% (w/v) Tween 80 to prevent bacterial aggregation. All bacterial strains were cultivated at 37°C with shaking in BD Falcon conical tubes or in conical flasks. Where needed, antimicrobials were added at the following concentrations (µg ml^−1^): hygromycin 50; kanamycin 50; tetracycline 0.02.

Bacterial growth was followed by measurement of absorbance at 580 nm in a Jenway spectrophotometer. Growth of *M. smegmatis* in various media was performed using a Bioscreen Growth Analyser as described previously [34]. Presented Bioscreen data are mean values of two independent experiments done in quintuplicate. Three independent transformants were used for all growth experiments. Viability was assayed by estimation of colony-forming unit (CFU) on 7H10 agar (Becton, Dickinson and Company). Supernatants were prepared from logarithmic phase cultures of *M. tuberculosis* (OD_580_ = 0.6–0.8) grown in Sauton's medium, filter sterilized twice and used for experiments immediately. For dose-dependency experiments culture supernatants were freeze-dried and reconstituted in sterile water. For low-magnesium control experiments concentration of MgSO_4_ in Sauton's medium was 0.2 mM.

### 2.2. Microscopy

*Mycobacterium smegmatis* bacilli were mounted on PBS and observed by phase contrast microscopy using a Diphot 300 inverted microscope with a 100 W mercury light source. Images were recorded using a 12/10 bit, high-speed Peltier-cooled CCD camera (FDI, Photonic Science) using Image-Pro Plus (Media Cybernetics) software. Labelling of nascent peptidoglycan was done as described previously [35]. Briefly, mycobacteria were grown in Sauton's medium in the presence of 20 ng ml^−1^ of tetracycline to exponential growth phase (OD_600_ ∼ 0.5). A mixture of Vancomycin-BODIPY (Life Technologies) and unlabelled vancomycin (1 : 1) was added to cultures to the final concentration of 2 µg ml^−1^, followed by a 2 h incubation at 37°C with shaking. Bacteria were washed twice with PBS containing 0.1% Tween 80 and used for fluorescence microscopy.

### 2.3. Generation PknB constructs for overexpression studies

The *pknB* gene and its variants (electronic supplementary material, table S1) were amplified from *M. tuberculosis* H37Rv DNA and cloned into the *Bam*HI and *Spe*I sites of the pMind plasmid [36] (for details of primers see electronic supplementary material, table S2). Constructs were confirmed by sequencing and electroporated into *M. smegmatis* or *M. tuberculosis*. Overexpression of *pknB* or its variants was induced by the addition of tetracycline and confirmed by qRT-PCR.

### 2.4. Preparation of muropeptides

Peptidoglycan from *Escherichia coli* and mycobacteria was isolated and purified as described previously [37,38]. For growth assays peptidoglycan (2 mg) was digested with mutanolysin, lysozyme, RpfB or MltA at 37°C for up to 72 h. Muramidases were used at the final concentration of 100 μg ml^−1^. Peptidoglycan digestion with mutanolysin and MltA was carried out in buffer containing 80 mM NaH_2_PO_4_, pH 4.8; with lysozyme in 25 mM NaH_2_PO_4_, pH 6.0; and with RpfB in 40 mM sodium citrate, pH 6.5. The presence of muropeptides was confirmed by HPLC. In separate experiments, peptidoglycan was sonicated three times for 30 s, briefly spun down at 100*g* to remove big fragments and used for growth assays. Synthetic muropeptides were prepared as described previously [39].

### 2.5. Determination of minimum inhibitory concentration for selected antimicrobials

Tests were performed in 96-well microtitre plates using a dilution method [40]. Briefly, *M. smegmatis* (1 × 10^5^ CFU) was inoculated in 100 µl of Sauton's medium supplemented with kanamycin and tetracycline, and a range of concentrations of antimicrobial tested. Plates were incubated for three days at 37°C. MIC was determined as the lowest concentration at which no visible growth was detected after 3 days of incubation. Fresh antimicrobial stocks were prepared for each experiment; four independent experiments were done.

### 2.6. Effect of recombinant PASTA protein on growth of *Mycobacterium tuberculosis*

Recombinant PASTA was purified as described previously [31] and filter sterilized prior to growth experiments. Mycobacteria (10^3^ cells ml^−1^) were inoculated in 16-well microtitre plates containing supplemented 7H9 medium with different concentration of sterile rPknB_PASTA. For each concentration four replicates were inoculated. Sealed plates were incubated at 37°C without shaking. Optical density was measured after 15 and 25 days of incubation. The experiment was repeated twice. Similar results were obtained when mycobacteria were grown in Falcon tubes or conical plastic flasks. *M. tuberculosis* stationary phase culture was stored at 4°C for two months and 2 × 10^4^ cells ml^−1^ were inoculated in conical flasks. The flasks were incubated at 37°C for up to 12 weeks. Optical density was measured at regular intervals using a Jenway spectrophotometer. The assay was performed in triplicate, three times.

### 2.7. Transcriptional profiling

Total RNA was isolated from 10 ml of mycobacterial cultures from exponential growth phase using the TRIzol method [41]. DNA contamination was removed with Turbo DNA-free DNAase (Ambion) before cDNA was generated using Superscript Reverse Transcriptase II (Invitrogen) and gene-specific primers (electronic supplementary material, table S2). Q-PCR was performed in a Corbett Rotor Gene 6000 real-time thermocycler using Absolute QPCR SYBR Green mix (Thermo) and gene-specific primers. Levels of expression were normalized to *16 s rRNA* [42].

### 2.8. Protein electrophoresis and Western blot

Mycobacteria were collected by centrifugation, washed in PBS and resuspended in buffer containing 50 mM Tris–HCl, pH 8.0 and 150 mM NaCl. Bacteria were lysed in FastPrep-24 Instrument (MP Biomedicals, UK) using glass beads. Lysates were centrifuged at 14 000*g* for 20 min to separate soluble and insoluble fractions. Proteins were separated, using 12% SDS PAGE and transferred on nitrocellulose membrane. Primary antibodies were anti-polyhistidine antibodies (Sigma); alkaline-phosphatase conjugate anti-mouse antibodies were used as secondary antibodies. Sigma Fast BCIP/NBT was used as a substrate to visualize recognized proteins.

### 2.9. Isolation of membrane protein fraction for proteomics analysis

Membrane fractions were prepared as described previously [43]. Briefly, *M. smegmatis* TMP and MIND strains were inoculated in 2 l conical flasks containing 500 ml Sauton's medium. The cultures were incubated at 37°C with shaking (200 r.p.m.) for 3 h. Cultures from two flasks were used for preparation of membrane fractions of each strain. The bacteria were harvested by centrifugation at 6000*g* for 20 min and washed with PBS. The pellets were resuspended in 10 ml of extraction buffer containing 50 mM Tris–HCl, pH 8.0, 150 mM NaCl, 10 mM MgCl_2_ and stirred on ice for 10 min. The cell suspension was sonicated before adding 100 µg of DNase I per gram of cells and stirring on ice for 5 min to decrease viscosity. The suspension was centrifuged at 25 000*g* for 25 min at 4°C. The pellet was discarded and the supernatant centrifuged at 100 000*g* for 60 min at 4°C. After discarding supernatant the pellet was carefully resuspended in 100 mM sodium carbonate buffer, pH 11.5 and centrifuged at 100 000*g* for 60 min. The centrifugation and resuspension in carbonate buffer steps were repeated a minimum of three times before a final resuspension and centrifugation step using sterile MilliQ water. The membrane fractions were resuspended in 1 ml sterile deionized water and freeze-dried.

### 2.10. Analysis of membrane proteins

Proteomics was carried out by the University of Leicester Proteomics Facility (PNACL, University of Leicester, http://www2.le.ac.uk/colleges/medbiopsych/facilities-and-services/cbs/protein-and-dna-facility/pnacl). Trypsin digestion of membrane proteins was performed using a filter-aided sample preparation method [44]. LC–MS/MS was carried out using an RSLCnano HPLC system (Dionex, UK) and an LTQ-Orbitrap-Velos mass spectrometer (Thermo Scientific). Peptides were eluted from the trap column at a flow rate of 0.3 µl min^−1^ and through a reverse-phase PicoFrit capillary column (75 μm i.d. × 400 mm) containing Symmetry C18 100 Å media (Waters, UK) that was packed in-house using a high-pressure device (Proxeon Biosystems, Denmark) over a period of 4 h, with the output of the column sprayed directly into the nanospray ion source of the LTQ-Orbitrap-Velos mass spectrometer.

The raw data file obtained from each LC–MS/MS acquisition was processed using Proteome Discoverer (v. 1.4.0.288, Thermo Scientific), searching each file in turn using Mascot [45] (v. 2.2.04, Matrix Science) against the UniProtKB-Swissprot database. The peptide tolerance was set to 10 ppm and the MS/MS tolerance was set to 0.02 Da. Fixed modifications were set as carbamidomethyl (C) and variable modifications set as oxidation (M). A decoy database search was performed. The output from Proteome Discoverer was further processed using Scaffold Q + S^4^ (v. 4.0.5, Proteome Software, Portland, OR, USA). For quantitative experiments the digested peptides were labelled with the TMTsixplex Label Reagent kit (Life Sciences) according to the manufacturer's instructions. Scaffold Q+ (v. 4.3.4, Proteome Software) was used to quantify Label Based Quantitation (iTRAQ, TMT, SILAC) peptide and protein identifications. Both protein and peptide identifications were accepted if they could be established at greater than 95.0% probability and contained at least two identified peptides. Peptide probabilities from X! Tandem were assigned by the Peptide Prophet algorithm [46] with Scaffold delta-mass correction. Peptide probabilities from Mascot were assigned by the Scaffold Local FDR algorithm. The mass spectrometry proteomics data have been deposited to the ProteomeXchange Consortium via the PRIDE partner repository with the dataset identifiers PXD002120 and 10.6019/PXD002120.

### 2.11. Analytical gel-filtration

Fifty microlitres of PknB_PASTA at a concentration of 50 µM were injected on a S75 10/300 column (GE Life Science) at a 0.4 ml min^−1^ flow rate. The column was equilibrated with 25 mM Tris–HCl (pH 8.5), and 100 mM NaCl with or without 25 mM MgSO_4_. For the injection in the column in the buffer without MgSO_4_, the protein was pre-incubated with 5 mM EDTA. In the case of gel-filtration in the presence of MgSO_4_, the protein was pre-incubated with 25 mM MgSO_4_.

### 2.12. Nuclear magnetic resonance experiments

All nuclear magnetic resonance (NMR) experiments were carried out at 20°C on Bruker Avance III 700 (^1^H–^15^N double resonance experiments) or Avance III 500 (^1^H–^13^C–^15^N triple-resonance experiments) spectrometers equipped with 5 mm z-gradient TCI cryoprobe, using the standard pulse sequences. NMR samples consist of approximately 50 µM ^15^N-labelled protein dissolved in 10 mM phosphate buffer (pH 6.8), 100 mM NaCl, with 5% D_2_O for the lock prepared as described elsewhere [31].

## 3. Results

### 3.1. The external PASTA domain is important for PknB function

It has been previously shown that overexpression of enzymatically active PknB in mycobacteria results in growth inhibition and alteration of bacterial shape [4]. Additionally, the external PknB_PASTA domain is indispensable for functional complementation of PknB conditional mutants of *M. smegmatis* and *M. tuberculosis* [47]. However, the precise role of the PknB_PASTA domain in regulation of mycobacterial growth and peptidoglycan biosynthesis remains unclear. In our initial experiments, we investigated growth patterns of *M. smegmatis* overexpressing *pknB*.

**Figure 2.**
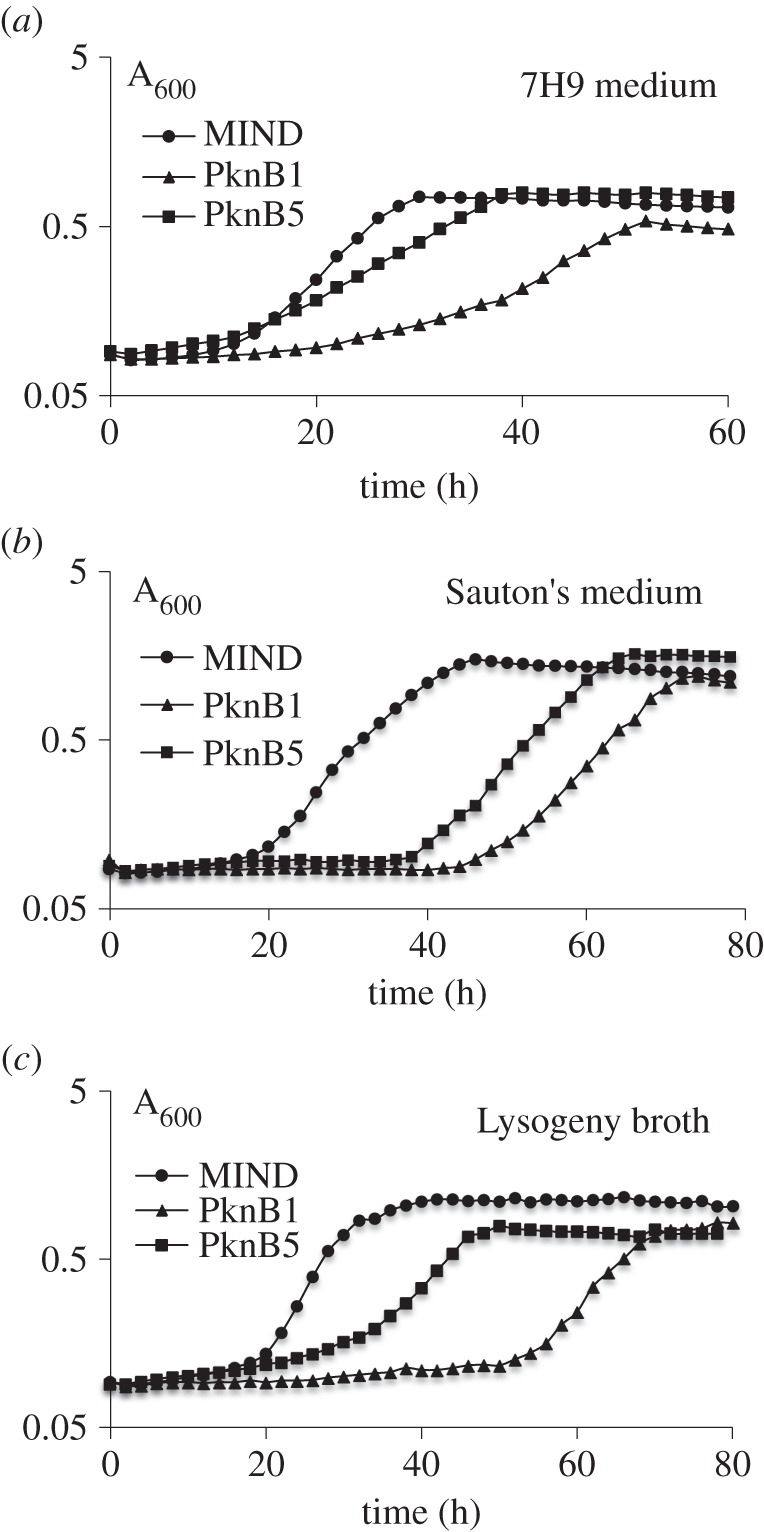
Effect of *pknB* and *pknB*-*ΔPASTA* overexpression on growth of *M. smegmatis*. Mycobacteria (5 × 10^6^) from early stationary phase were inoculated in 100-well honeycomb plates, containing various media and incubated in Bioscreen Growth analyser at 37**°**C with shaking for 5 days in the presence of tetracycline. Presented data are mean values of 5 replicates from two independent experiments. Standard deviations were 10% or less of average values and are not shown for clarity.

A full-length *pknB* or its truncated form missing the region encoding the external PASTA domain (*pknB-ΔPASTA*) was cloned into pMind plasmid [36], containing a tetracycline-regulated promoter (figure 1; electronic supplementary material, table S1). The plasmids were transformed into *M. smegmatis* and the resultant strains, designated as PknB1 (*pknB*), PknB5 (*pknB-ΔPASTA*) and MIND (empty plasmid), grew similarly in all media tested in the absence of the inducer tetracycline (data not shown). However, addition of tetracycline to the PknB1 or PknB5 strains affected growth in liquid medium (figure 2). This inhibitory effect was probably caused by the induction of expression of *pknB* and *pknB-ΔPASTA* from the pMind plasmid. When grown in the presence of tetracycline, PknB1 and PknB5 expressed the *pknB* versions from pMind at relatively high level, corresponding to 1% (±0.3%) of all *16s-rRNA* transcripts as judged by quantitative RT-PCR; no expression of the target gene was detected in mycobacteria in the absence of tetracycline. Interestingly, overexpressing the *pknB* versions resulted in different growth characteristics. In all media, the PknB1 strain had a longer apparent lag phase (time to detectable turbidity) and slower growth rate (figure 2; electronic supplementary material, table S3), indicating that overexpression of the truncated PknB missing the external PASTA domain was less toxic than overexpression of the full-length kinase.

**Figure 3.**
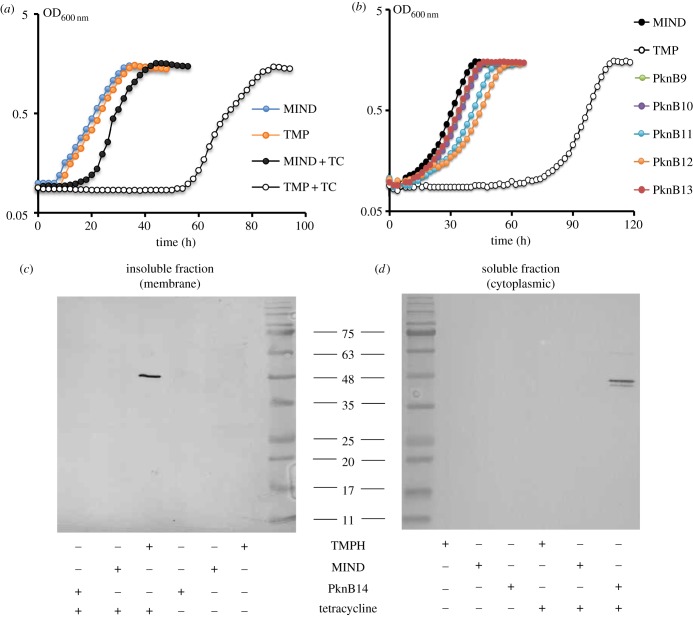
Effect of PknB_PASTA overexpression on *M. smegmatis* growth*.* (*a*) Comparative growth kinetics of *M. smegmatis* strains in Sauton's medium. The same inoculum (approx. 2 × 10^6^ cells per well) was used to seed induced (+tetracycline) and control cultures, respectively. (*b*) Overexpression of non-secreted PASTA domains or transmembrane domain on its own does not affect mycobacterial growth. (*c*) Detection of PknB_TM-PASTA-6 × HIS in membrane fraction of *M. smegmatis* strains by western blotting. (*d*) Detection of the PASTA domain missing the transmembrane region in cytoplasmic fraction of *M. smegmatis* strains by Western blotting.

To further investigate the role of the PknB_PASTA domain in bacterial growth directly, we cloned the *pknB* region encoding the membrane-anchored external domain of *M. tuberculosis* (designated as *tmp*, figure 1) into the pMind plasmid and transformed this plasmid into *M. smegmatis*. In the absence of tetracycline, both MIND and TMP strains grew similarly (figure 3*a*, filled symbols) and their cDNA analysed by q-RT-PCR contained similar levels of *tmp* transcripts. However, addition of tetracycline led to overexpression of the *tmp* transcript (reaching 9.6 ± 0.8% from the total 16 s *rRNA* transcripts) in the TMP strain, which resulted in growth inhibition, especially in Sauton's medium (electronic supplementary material, table S3; figure 3*a*, open symbols). The TMP strain required a longer time to initiate bacterial growth, but its growth rate was identical to the one observed in the MIND control strain. This phenotype was highly reproducible and did not change after passage *in vitro* (electronic supplementary material, figure S1). Expression of the PASTA domain without the transmembrane domain (PknB13), the transmembrane domain on its own (PknB9) or the transmembrane domain with one PASTA unit (PknB10; figure 1) had no significant effect on bacterial growth (figure 3*b*). Strains overexpressing the PASTA domain with two or three PASTA units (PknB11 and PknB12, respectively) displayed a marginal growth defect. All PASTA variants were expressed at similar levels according to qRT-PCR (data not shown). We generated Myc-6xHis-tagged versions of TMP (designated as TMPH strain; electronic supplementary material, table S1) and of the soluble PASTA domain (designated as PknB14) and investigated their localization by western blot. TMPH was detected in the envelope fraction, while PknB14 was found in the cytoplasm *M. smegmatis* (figure 3*c,d*). Importantly, TMPH and TMP strains showed no difference in growth under all conditions described here (data not shown). These results confirm that only the surface-exposed domain containing four PASTA units significantly impaired mycobacterial growth.

**Figure 4.**
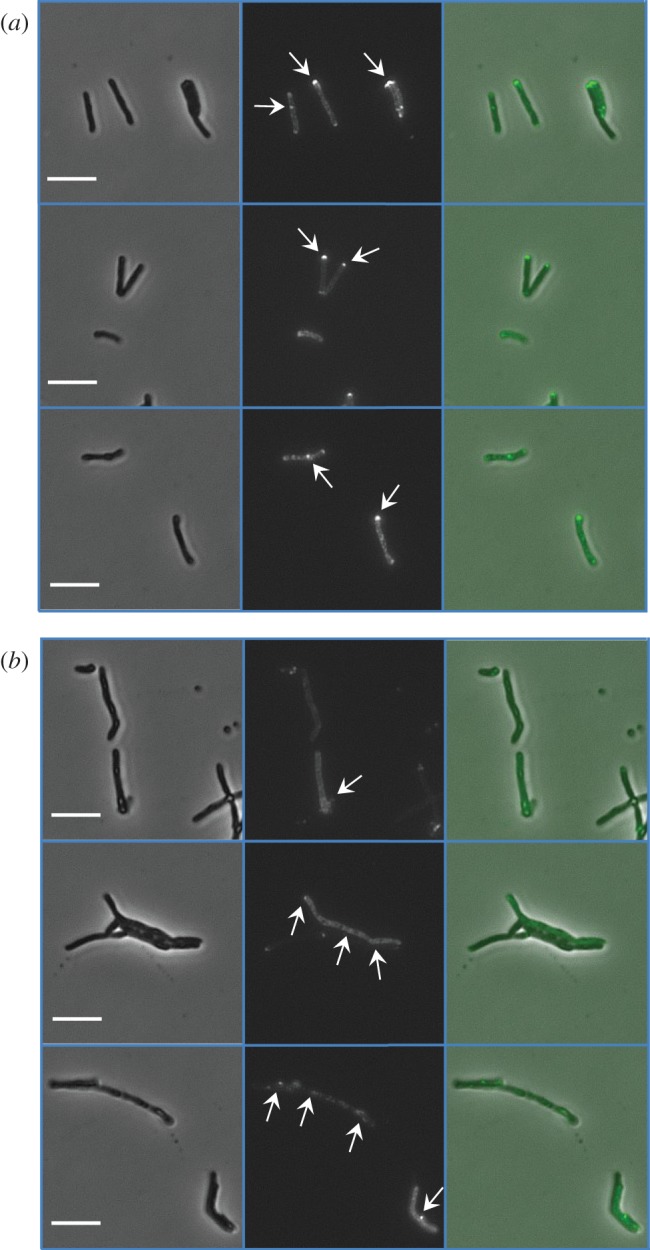
Overexpression of PknB_PASTA results in alteration of bacterial morphology and vancomycin labelling. Mycobacteria from logarithmic growth phase were labelled with Vancomycin-BODIPY conjugate, washed and mounted in PBS and examined using a Diphot 300 inverted microscope. Three independent fields for each strain are shown. Bars indicate 5 μm. (*a*) MIND and (*b*) TMP. Left panel, phase contrast; middle panel, fluorescence microscopy; right panel, merged images. Arrows indicate labelled peptidoglycan.

Overproduction of the PknB_PASTA domain also influenced cellular morphology. Microscopic examination of the strains revealed accumulation of elongated TMP cells of irregular shape (figure 4*b*, left panel), similar to cells with partially depleted PknB [4], suggesting that the overexpressed PASTA domain possibly interfered with the PknB function. Furthermore, Vancomycin-BODIPY labelling revealed anomalous distribution of nascent peptidoglycan in TMP mycobacteria (figure 4*b*, middle panel). While in control MIND cells newly synthesized peptidoglycan was mainly localized at the poles and mid-cell (figure 4*a*), TMP cells displayed a diffused staining across the entire cell surface (figure 4*b*). We also observed significant proportions of unlabelled or weakly labelled TMP cells (figure 4*b*). TMP mycobacteria tend to aggregate, suggesting a modified cell surface, delayed cell separation or increased lysis.

### 3.2. Elevated Mg^2+^ ion level eliminates TMP-mediated growth inhibition

**Figure 5.**
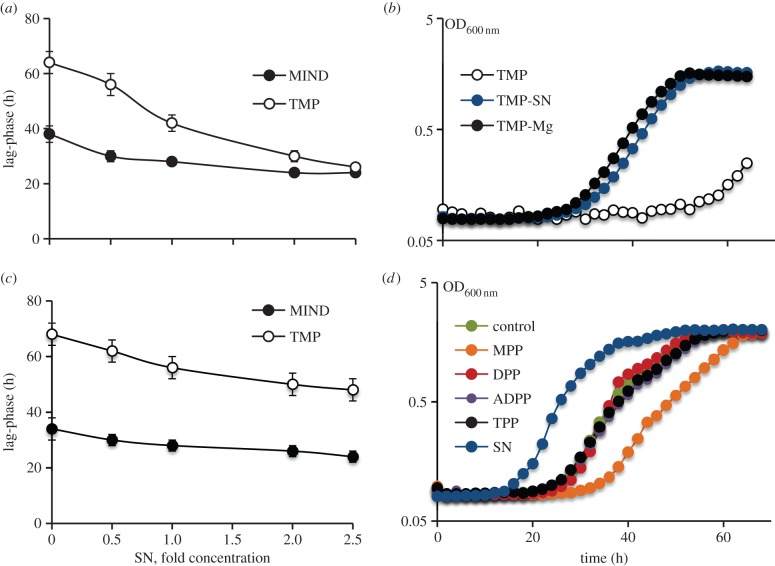
Culture supernatant and high concentration of Mg^2+^ abolish the inhibitory effect caused by PknB-PASTA overexpression. (*a*) Culture supernatants abolish growth-inhibitory effect of PknB_TM-PASTA overexpression in a dose-dependent manner. Relative concentration of culture supernatant added is expressed as ‘fold concentration’. A value of onefold corresponds to undiluted culture supernatant. The apparent lag phase was calculated as a period of time when culture reached OD_600 nm_ 0.1. (*b*) Mg^2+^ (10 mM) relieves growth inhibition of TMP in a manner comparable with undiluted culture supernatant. (*c*) Effect of low Mg^2+^ supernatant on apparent lag phase of TMP strain. (*d*) Effect of synthetic muropeptides on growth of *M. smegmatis*. Mycobacteria from early stationary phase were washed in Sauton's medium twice and 5 × 10^6^ bacteria were inoculated in Sauton's medium, containing culture supernatant or synthetic muropeptides at final concentration of 10 µM. MPP, MurNAc-pentapeptide; DPP, GlcNAc-MurNAc-peptapeptide; ADPP, GlcNAc-1,6 anhydromuramyl-pentapeptide; TPP, tetra-saccharide-peptide; SN, 50% (v/v) culture supernatant. GlcNAc, *N*-acetylglucosamine; MurNAc, *N*-acetylmuramic acid; pentapeptide, l-Ala-γ-d-Glu-*m*-DAP-d-Ala-d-Ala. (*b*,*d*) Presented data are mean values of 5 replicates from two independent experiments. Standard deviations were 10% or less of average values and are not shown for clarity. SN, media were supplemented with culture supernatant.

The results described above support the functional importance of the PknB_PASTA domain for PknB activity. The structural studies of the PknB_PASTA suggested that this domain could dimerize beacuse of conformational flexibility between the different PASTA domains [31]. It was proposed that the binding of putative ligands could induce the dimerization of kinase domains and consequent trans-activation of PknB [31]. Thus, the PASTA domain could serve as a receptor for signalling molecules (e.g. muropeptides) and activate bacterial resuscitation and growth, employing the mechanism previously proposed for *B. subtilis* spore germination [16,39]. The PknB_PASTA domain missing the functional kinase domain may compete with the native PknB for the ligands and dysregulate the PknB-mediated signalling pathways. Therefore, we investigated whether culture supernatant from growing mycobacteria (potentially containing the putative ligand) would eliminate the growth defect caused by TMP overexpression. Indeed, the growth inhibition was abolished by the addition of culture supernatant in a dose-dependent manner (figure 5*a*). Further tests showed that the active compound was a low-molecular-weight chemical resistant to heating and it was present in Sauton's medium prior to exposure to bacteria (data not shown). Eventually, we demonstrated that the active entity was Mg^2+^ from MgSO_4_ or MgCl_2_. Elevated concentration of Mg^2+^ could completely abolish the inhibition by PASTA-domain overexpression (figure 5*b*) and the dose-dependent effect of conditioned medium was largely because of increased Mg^2+^ concentrations (data not shown). Supernatants obtained from cultures grown in lower-MgSO_4_ (0.2 mM) medium had only a moderate growth-stimulatory effect on both control and TMP strains (figure 5*c*). The increased Mg^2+^ concentration did not influence expression of *tmp* (*p* > 0.05, *t*-test) and the production of TMPH, a His-tagged version of TM-PASTA (data not shown). Magnesium also improved growth of PknB1 and PknB5 strains (data not shown).

To investigate whether the PknB_PASTA domain was able to bind Mg^2+^ ions, we used NMR methodology [31]. A ^15^N-labelled sample of recombinant PknB_PASTA (50 µM) was titrated with increasing amounts of MgCl_2_ (or MgSO_4_) up to 50 mM. The ion binding with either a lateral chain or the protein backbone would generate chemical-shift perturbation on the ^1^H–^15^N resonance measured in the HSQC experiment [48]. However, no chemical-shift perturbation was observed in the PknB_PASTA upon addition of MgCl_2_ or EDTA, suggesting a lack of interaction between the protein and Mg^2+^ ions (electronic supplementary material, figure S2), and the addition of MgSO_4_ to recombinant PASTA did not change the oligomerization state of recombinant PASTA (electronic supplementary material, figure S3). These results show that the ameliorating effect of Mg^2+^ on TMP-mediated growth inhibition was indirect.

### 3.3. Muropeptides do not eliminate TMP-mediated growth inhibition

In separate experiments, we tested the effects of muropeptides on the growth inhibition in the TMP strain. We used peptidoglycan from *E. coli* digested with various muramidases, sonicated or RpfB-digested peptidoglycan from *M. smegmatis*, and synthetic muropeptides, *N*-acetylglucosamine, *N*-acetylmuramic acid and *N*-acetylglucosaminyl–*N*-acetylmuramic acid (electronic supplementary material, table S4). We only observed a minor reduction in the duration of lag phase of TMP strain from 65 to 52 h in the presence of relatively high concentrations of digested or sonicated peptidoglycan (0.5 mg ml^−1^ and higher). The observed reduction in lag phase did not depend on the type of peptidoglycan used. Synthetic muropeptides, previously employed for germination of *B. subtilis* spores [39], did not reduce growth inhibition at any concentration tested (10 pM–50 mM) and did not stimulate growth of *M. smegmatis* (figure 5*d*). Among other divalent metals tested Ca^2+^ improved the TMP growth; however, it could not be used at concentrations above 5 mM because of poor solubility. Since Mg^2+^ ions are known to stabilize the cell wall and are frequently used for cultivation of bacteria with defective cell walls [49], we investigated whether the TMP strain was more susceptible to various antimicrobials compared with control.

### 3.4. Overexpression of PknB-PASTA increases sensitivity of *Mycobacterium smegmatis* to β-lactam antibiotics and alters the abundance of cell-wall proteins

We tested minimum inhibitory concentrations (MIC) of various antimicrobial agents for PknB1, TMP and MIND strains. MIC of rifampicin (inhibition of transcription), ethambutol (arabinogalactan biosynthesis) and streptomycin (protein biosynthesis) were identical in all the strains tested (table 1). However, the TMP strain was remarkably more sensitive to several β-lactam antibiotics: meropenem, ampicillin and clavulanate, an inhibitor of β-lactamase. In the presence of sub-inhibitory concentration of clavulanate (100 μM), MIC of meropenem for the TMP strain decreased below 0.15 μM compared with 6.5 μM in the control strain. Interestingly, overexpression of PknB did not alter antimicrobial susceptibility, suggesting that the PknB-PASTA domain on its own may perturb peptidoglycan synthesis, causing higher sensitivity to β-lactams. The perturbation of peptidoglycan synthesis could also explain the prolonged lag phase observed in the TMP strain. To address this possibility, membrane proteins isolated from the MIND and TMP strains in lag phase were investigated.

**Table 1.** Effect of PknB-PASTA overexpression on antimicrobial susceptibility of *M. smegmatis.*

strain	minimum inhibitory concentration against antibiotic (µM)						
	rifampicin	streptomycin	ethambutol	meropenem	clavulanate	meropenem+clavulanate^a^	ampicillin
MIND	1.2	0.4	2.5	13–19.5	>1000	6.5–13.0	140–170
PknB1	1.2	0.4	2.5	13–19.5	>1000	6.5–13.0	140–170
TMP	1.2	0.4	2.5	6.5	150–200	0.13	30–45

^a^Clavulanate was added at a concentration of 100 µM.

In total, we identified 1071 proteins in the membrane fractions of both strains using LTQ-Orbitrap-Velos mass spectrometry. The TMPH strain had 90 proteins specific for this sample and 95 proteins were detected in the MIND strain only. The majority of proteins (886) were identified in both strains. Importantly, production of the PknB_PASTA domain in the membrane fraction was confirmed by detection of 22 unique fragments with 86% peptide coverage in the TMPH strain (electronic supplementary material, table S5). Most of the proteins detected (705) were assigned as hypothetical with unknown function, while 267 were predicted as membrane or cell-surface proteins. This result confirms that the extraction procedure selectively enriched the membrane proteins. A high number of membrane proteins implicated in transport of metals, nutrients and enzymes was found in the membrane fractions of both strains. Next, peptides were labelled using the TMPsixplex kit to compare the relative abundance of membrane proteins in TMP and MIND (electronic supplementary material, table S5). We focused our analysis on cell division proteins and enzymes involved in cell-wall biosynthesis. Most cell division proteins were equally present in the TMP and MIND strains (data not shown). However, CwsA was more abundant in the TMP strain (table 2). The precise function of CwsA is unknown but it has been shown to interact with CrgA, which is annotated as an inhibitor of septum formation [50,51]. CrgA was detected only in the membrane fractions of TMP but not in those of MIND, and its relative abundance could not be calculated. Another CrgA interaction partner, Wag31, was detected at similar levels in both strains. Interestingly, the TMP strain had more enzymes involved in biosynthesis of mycolyl-arabinogalactan (table 2; electronic supplementary material, table S5), including several mycolyl- and galactofuranosyl-transferases. Regarding peptidoglycan-related enzymes, the levels of two metallo-β-lactamases, l,d-transpeptidase LdtB and d-alanyl-d-alanine carboxypeptidase DacB1, were increased in the TMP membrane fraction (table 2). Complete data are available via ProteomeXchange with identifier PXD002120. Collectively these results may suggest that the overexpression of the PASTA domain influences the biosynthesis and architecture of mycobacterial cell wall.

**Table 2.** Cell-wall enzymes and proteins differently abundant in TMP compared with MIND.

*M. tuberculosis* protein	*M. smegmatis* protein	protein description	ratio TMP/MIND log2-fold
Rv0014c	MSMEG_0028	His-Tagged TM-PASTA	5.9
Rv0008c	MSMEG_0023	cell-wall protein CwsA	1.0
Rv3804c	MSMEG_6398	FbpA mycolyl transferase	1.0
Rv3790	MSMEG_6382	DprE1 decaprenylphosphoryl-β-d-ribose 2′oxidase	0.9
Rv3577	MSMEG_6071	metallo-β-lactamase	0.9
Rv0129c	MSMEG_3580	FbpB mycolyl transferase	0.6
Rv3782	MSMEG_6367	galactofuranosyl transferase	0.8
Rv3808c	MSMEG_6403	galactofuranosyl transferase	0.8
Rv3265	MSMEG_1826	dTDP-RhA:a-d-GlcNAc-diphosphoryl polyprenol, a-3-l-rhamnosyl transferase	0.7
Rv0906	MSMEG_5638	metallo-β-lactamase	0.6
Rv2518c	MSMEG_4745	LdtB l,d-transpeptidase	0.6
Rv0237	MSMEG_0361	glycosyl hydrolase	0.6
Rv3330	MSMEG_1661	DacB1D-alanyl-d-alanine carboxypeptidase	0.5
Rv2748c	MSMEG_2690	DNA translocase FtsK	0.5
Rv2171	MSMEG_4239	conserved lipoprotein	−0.8
Rv2721c	MSMEG_2739	transmembrane alanine and glycine-rich protein	−0.5

### 3.5. The addition of recombinant PASTA protein abolishes growth of *Mycobacterium tuberculosis*

**Figure 6.**
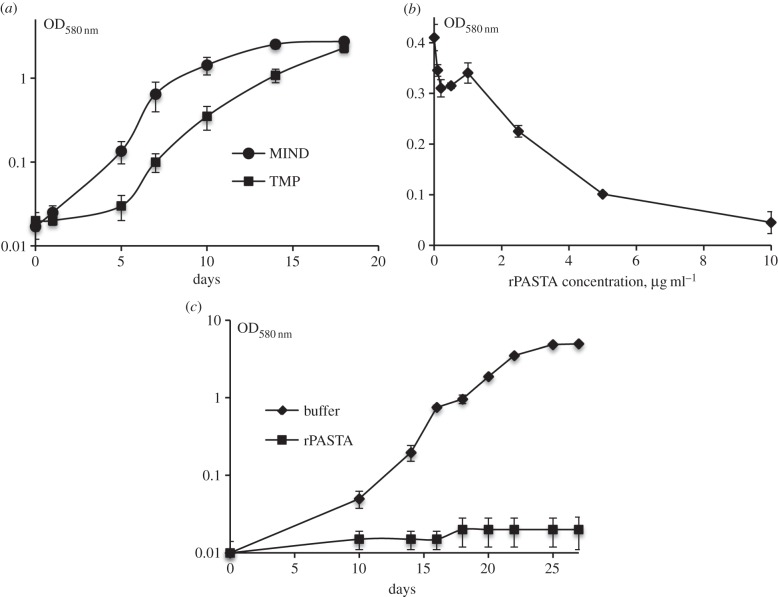
PknB_PASTA reduces growth of *Mycobacterium tuberculosis*. (*a*) Overexpression of PknB_PASTA domain delays growth initiation. Mycobacteria (approx. 1 × 10^6^) were inoculated in supplemented 7H9 medium containing hygromycin and tetrocycline. (*b*) Effect of different concentrations of rPASTA on *M. tuberculosis* growth. Mycobacteria (10^3^ cells ml^−1^) were inoculated in supplemented 7H9 medium containing different concentration of sterile rPASTA. The optical density was measured after 21 days of incubation. (*c*) Regrowth of stored *M. tuberculosis* was delayed by the addition of rPASTA. *M. tuberculosis* (2 × 10^4^ cells ml^−1^) was inoculated in supplemented 7H9 medium. Buffer was used as control and rPASTA was added at final concentration of 10 µg ml^−1^. (*b*,*c*) Each data point is an average value of 3 biological replicates, error bars indicate standard deviations. Experiments were performed three times and results of one typical experiment are shown.

The results described above suggest that overexpression of the PknB_PASTA domain can be employed for growth inhibition and increase of susceptibility to antimicrobials in medically important pathogen *M. tuberculosis*. We therefore investigated whether the PknB_PASTA domain would have a similar effect in *M. tuberculosis*. We transformed pMind *pknB7* and empty pMind plasmids (electronic supplementary material, table S1) into *M. tuberculosis* H37Rv and investigated growth of the resultant strains (TMP and MIND) in the presence of tetracycline. As shown in figure 6*a*, overexpression of the external membrane attached PASTA (TMP) indeed inhibited initiation of *M. tuberculosis* growth. We next studied if addition of recombinant PASTA protein containing PASTA units only (residues 354–626, designated as rPASTA) may alter the growth. We noted that the addition of rPASTA to *M. tuberculosis* resulted in growth defect at concentration of 10 µg ml^−1^ (figure 6b) and increased apparent lag phase from 5 to 14 days. This growth inhibition was temporary and the cultures eventually produced normal growth, while addition of recombinant PASTA to logarithmic phase culture had no effect on growth (data not shown). However, the growth-inhibitory effect of rPASTA was more pronounced in experiments when mycobacteria were stored at 4°C for two months (figure 6c). We found that these cells retained culturability and produced normal growth both in liquid and solid media. Nevertheless, their growth initiation was significantly delayed by addition of rPASTA at concentration 10 µg ml^−1^. The mycobacteria were not killed by rPASTA and after two-month incubation generated normal stationary phase culture (figure 6*c*). In all experiments, control buffer did not have any inhibitory effect. These results suggest that the PASTA domain may directly interfere with mycobacterial regrowth and, therefore, presents a plausible target for the design of specific drugs altering bacterial regrowth and resuscitation.

## 4. Discussion and conclusion

PknB has been shown to regulate bacterial division, growth and cell-wall biosynthesis [10]. The data presented in this study suggest that the PknB-mediated signalling pathway can be targeted through the external PknB_PASTA domain. Overexpression of the PknB_PASTA domain delayed the regrowth of both *M. tuberculosis* and *M. smegmatis*. The growth inhibition was ameliorated by addition of high concentrations of Mg^2+^ but not by muropeptides. Mg^2+^ ions are crucial for all living organisms, and the concentration may vary, reaching as high as 20 mM in eukaryotic [52] and a massive 100 mM in prokaryotic cells [53]. In Gram-negative bacteria magnesium ions stabilize the outer membrane [54] and are found in high concentrations in the cell wall of Gram-positive bacteria, which bind divalent ions to their teichoic acids [54]. Mycobacteria do not possess teichoic acids and the binding of Mg^+2^ ions has not been studied in the mycobacterial cell wall in detail, although Mg^2+^ ions improved growth of mycobacteria in media with low pH [55]. Growth of *B. subtilis* mutants missing certain peptidoglycan binding proteins was improved at high Mg^2+^ levels, suggesting that divalent metals may compensate growth defects due to altered peptidoglycan biosynthesis [56].

**Figure 7.**
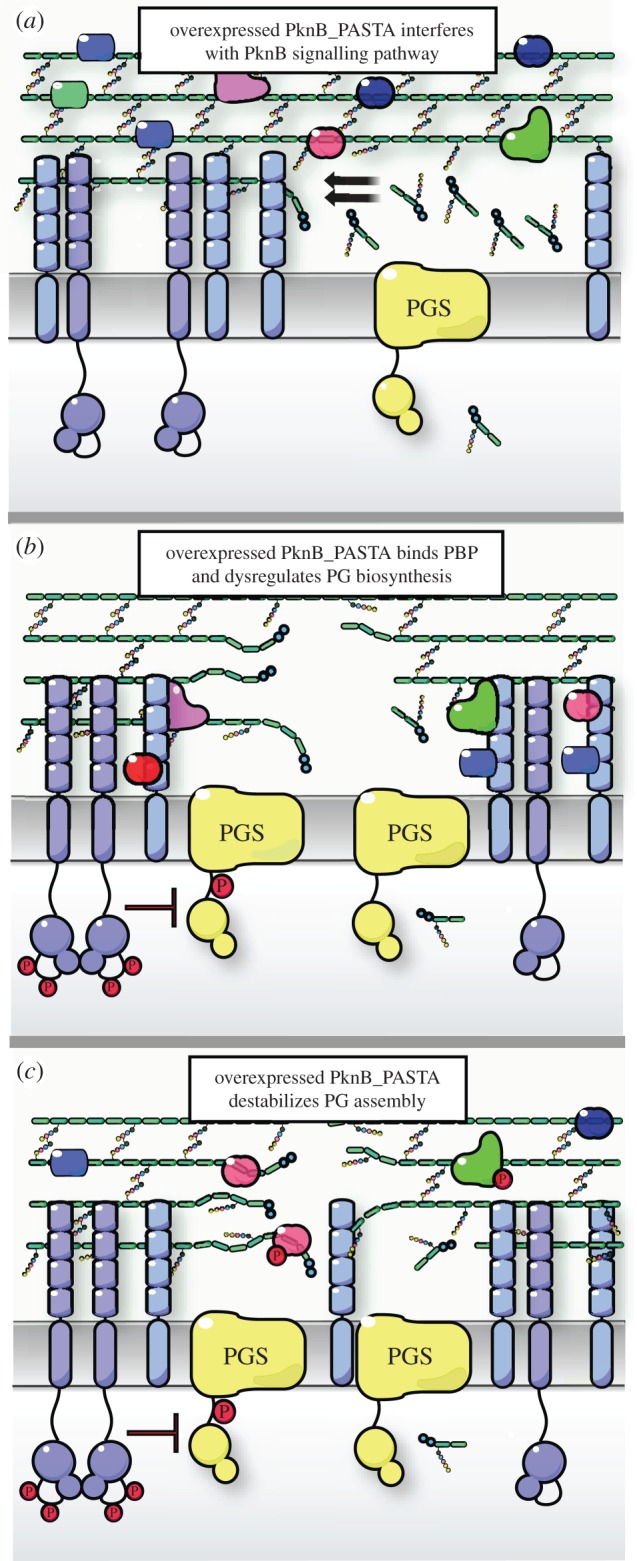
Schematic representation of possible mechanisms of PknB_PASTA-mediated growth inhibition. (*a*) In the TMP strain the overexpressed PknB_PASTA competes with native PknB for peptidoglycan ligands and interferes with PknB-mediated signalling. (*b*) Overexpressed PknB_PASTA binds penicillin-binding proteins and dysregulates peptidoglycan polymerization. (*c*) Overexpressed PknB_PASTA binds to peptidoglycan and destabilizes its structure. PGS, peptidoglycan synthesis complex.

The precise mechanism of the magnesium effect on growth of the TMP strain is unknown. Mg^2+^ ions may stabilize the cellular membrane and protein complexes associated with the membrane disturbed by overexpression of PknB_PASTA domain, although it is also possible that Mg^2+^ ions directly bind to the growing strands of stems of peptidoglycan and promotes their polymerization. Further dissection of molecular mechanisms underlying the inhibitory effect of PknB_PASTA overexpression will facilitate our understanding of the biological function of PknB and its extracellular domain, as well as the roles of Mg^2+^ ions in cell-wall architecture. Three possible scenarios may explain the observed effects associated with overexpression of PknB_PASTA (figure 7). (i) The overexpressed PknB_PASTA competes for the ligand (peptidoglycan) with a native PknB, and therefore interferes with the PknB-mediated signalling pathway (figure 7*a*). (ii) The PknB_PASTA domain interacts with penicillin-binding proteins and dysregulates peptidoglycan biosynthesis (figure 7*b*). (iii) Finally, highly abundant PknB_PASTA may directly disrupt the structure of peptidoglycan by binding to the growing strand, and physically prevent extension and cross-linking (figure 7*c*).

The regulatory function of PknB (phosphorylation of cell-wall enzymes and division factors) is evolved to coordinate cell-wall biosynthesis and division. In fact, a recently published study established that PknB coordinated assembly of peptidoglycan synthesizing complex via recruiting FhaA and phosphorylating MviN, a protein essential for mycobacterial growth [26].

In *Listeria monocytogenes* the inactivation of a PASTA-containing kinase, a homologue of PknB, resulted in increased susceptibility to β-lactam antimicrobials [57], as in the TMP strain, which presumably shows high susceptibility to β-lactam antimicrobials because of partial inactivation of PknB function. However, while the PknB mutant lyses and is not able to grow, the TMP strain only shows an initial impairment of growth in the form of a prolonged lag phase, but is able to grow normally in exponential phase. Thus, other factors apart from interference with PknB function may contribute to the growth phenotype of the TMP strain.

A possible direct interaction of PknB_PASTA with penicillin-binding proteins is in accordance with recent findings that the PASTA domain of StkP kinase from *S. pneumoniae* was able to bind penicillin-binding protein 2× [58]. We also cannot exclude that the PASTA domain changes the architecture of the cell wall by interacting directly with peptidoglycan (figure 7*c*), as was previously observed *in vitro* [16,59]. The results presented in this study may suggest that the overexpressed PknB_PASTA domain interacts with growing strands of peptidoglycan and possibly physically interferes with its cross linking (figure 7*c*), culminating in higher susceptibility of the overexpressing strain to meropenem, clavulanate and ampicillin. Meropenem has been shown to inhibit both dd-carboxypeptidase and ld-transpeptidase activities [60], and in combination with clavulanate it is highly active against growing and persisting mycobacteria [61].

Our findings are consistent with the previously proposed peptidoglycan-sensing role of PknB_PASTA [11,30]. Further investigation is required to clarify whether the PknB_PASTA domain recognizes the specific structures present in the region of peptidoglycan growth and therefore activates dimerization of the catalytic domains, or whether it plays a more structural role in supporting growing peptidoglycan and ensuring proper localization of PknB. Strains overexpressing truncated forms of the PASTA domain did not have pronounced growth defect (figure 3*b*), suggesting that four PASTA units and the transmembrane region are required for PknB functionality. Removal of the transmembrane part resulted in mislocalization of the PASTA domain. Although the precise role of the individual PASTA units awaits elucidation, our data are consistent with the previous observation that the truncated PknB missing one or more PASTA domains was not able to complement the conditional *pknB* mutant [47]. It is also remains unclear whether the PASTA domain can sense short muropeptides released by mycobacteria during growth. In our experiments, we obtained a very modest effect of peptidoglycan on the growth inhibition caused by PASTA overproduction and no stimulation of growth or resuscitation by externally added muropeptides. It is possible that specific muropeptides are required for activation of PknB and subsequent resuscitation, as muropeptides have been shown to possess resuscitation-stimulatory activity [62]. It is conceivable that the PASTA domain may be directly involved in resuscitation by sensing *in vivo* rearrangement of peptidoglycan, possibly because of the action of resuscitation-promoting factors [34,63] and other muramidases, and regulation of cell-wall biosynthesis. Additional experiments on monitoring rearrangement of peptidoglycan and interaction between the external PASTA domain and stem peptidoglycan, using solid-state NMR [64] and probes for microscopy of living bacteria such as highly sensitive atomic force microscopy cantilevers [65] will allow us to establish the precise PknB-mediated sensing mechanism.

## Supplementary Material

Turapov 2015 supplemental material figures revised

## Supplementary Material

Turapov 2015 supplemental material tables revised

## Supplementary Material

Table S5

## Data accessibility

The datasets supporting this article have been uploaded as part of the electronic supplementary material.
