# Supplementary material for: The external PASTA domain of the essential serine/threonine protein kinase PknB regulates mycobacterial growth
Source: Open Biol. 2015 Jul 1;5(7):150025. doi: 10.1098/rsob.150025 (PMC4632501; doi:10.1098/rsob.150025)
Supplement: Turapov 2015 supplemental material figures revised [file rsob150025supp1.pdf]

Supplemental Information

The external PASTA domain of the essential serine/threonine protein kinase PknB regulates mycobacterial growth

Obolbek Turapov[1], Jessica Loraine[1$], Christopher H. Jenkins[1], Philippe Barthe[2, 3], Daniel McFeely[1&], Francesca Forti[4], Daniela Ghisotti[4], Dusan Hesek[5], Mijoon Lee[5], Andrew R. Bottrill[1], Waldemar Vollmer[6], Shahriar Mobashery[5], Martin Cohen-Gonsaud [2, 3] and Galina V. Mukamolova[1]*

[1]Department of Infection, Immunity and Inflammation, University of Leicester, Leicester, LE1 9HN, UK; [2]Centre de Biochimie Structurale, CNRS UMR 5048, 29, rue de Navacelles 34090 Montpellier, France; [3]INSERM U1054, Université Montpellier I et II, Montpellier, France; [4]Dipartimento di BioScienze, Università degli Studi di Milano, Italy; [5]Department of Chemistry and Biochemistry, 423 Nieuwland Science Center, University of Notre Dame, Notre Dame, IN 46556 USA; [6] Centre for Bacterial Cell Biology, Institute for Cell and Molecular Biosciences, Newcastle University, Newcastle upon Tyne, NE2 4AX, United Kingdom.

Content

Table S1. Plasmids and strains generated for over-expression studies.

Table S2. Primers used in the study.

Table S3.Growth of *M. smegmatis* strains over-expressing *pkn*B variants.

Table S4. Muropeptides and sugars tested in TMP growth assays.

Table S5. List of proteins differently present in the membrane fractions of TMP and MIND (submitted as a separate excel file). Peptides from the TMP and MIND samples were labelled with different tandem mass tags. During MS/MS fragmentation each tag generates a reporter-ion with a different m/z value. The peaks areas of the reporter-ion can thus be compared to give a relative quantitative value. The data were normalized separately within each acquisition run. Intensities for each peptide identification were normalized within the assigned protein. The reference channel (MIND) was normalized to produce a 1:1 fold change. All normalization calculations were performed using medians to multiplicatively normalize data. The values shown thus correspond to the $\log_2$ fold change for the TMP sample normalized against the MIND (reference) sample.

Figure S1.

Figure S2.

Figure S3.

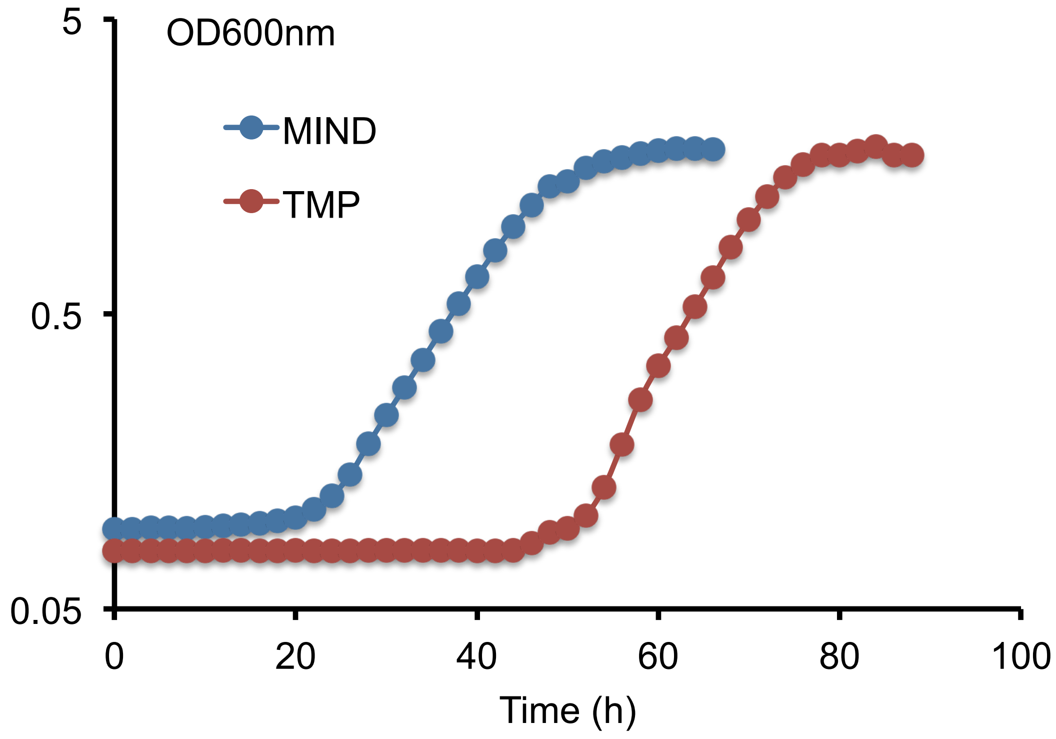

Figure S1. Growth of *M. smegmatis* MIND and TMP strains in Sauton's medium supplemented with hygromycin and tetracycline after *in vitro* passage. Mycobacteria were grown in Sauton's medium to stationary phase and used for inoculation of microtitre plates as described in Materials and Methods.

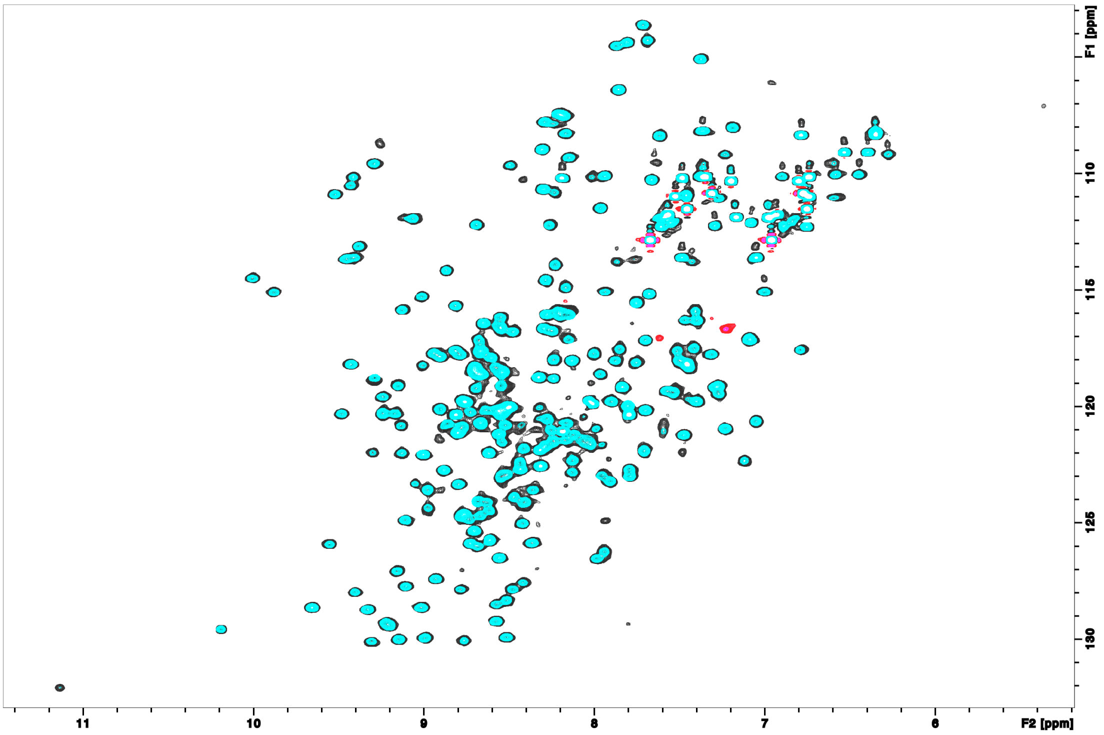

Figure S2. Superimposition of [^{1}H, ^{15}N] HSQC spectra from PknB_PASTA in 25 mM sodium acetate pH 4.6 in the presence (black) or absence (blue) of 25 mM $MgCl_2$, recorded at 37°C.

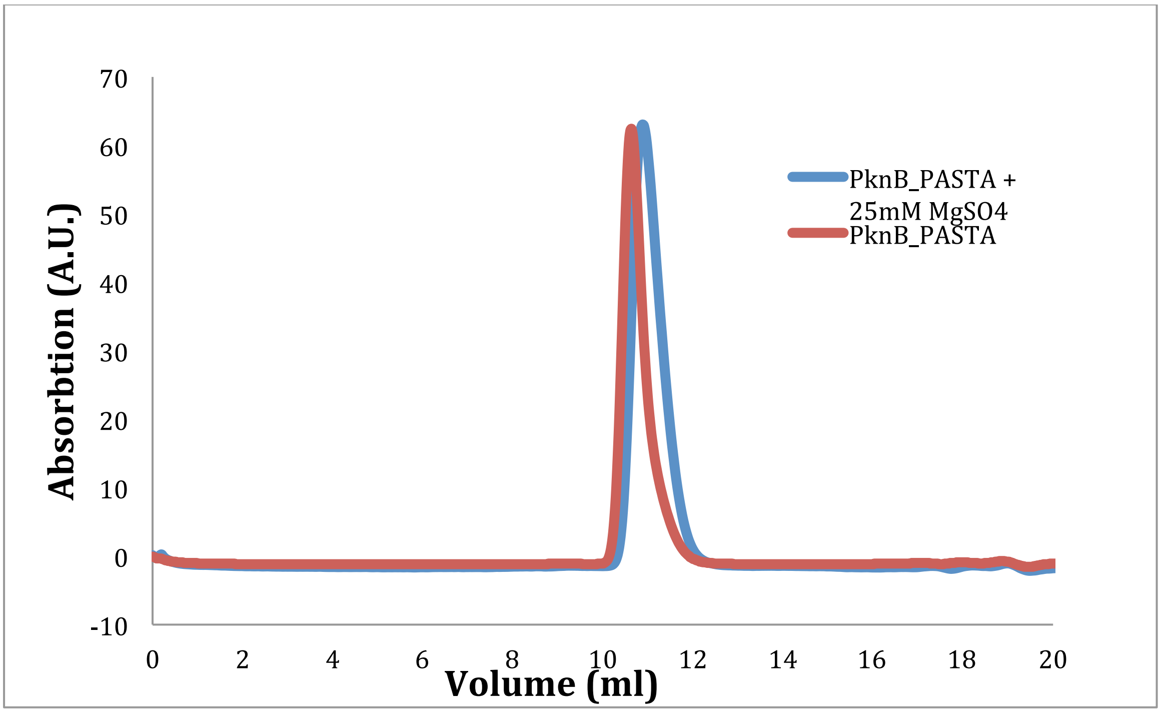

Figure S3. Analytical gel filtration chromatograms of PknB_PASTA (50 μl at 50 μM) with (blue) or without (red) 25 mM $MgSO_4$. Both profile are almost identical indicating that the protein is not subject to any major conformational changes or oligomerization in presence of a large excess of $MgSO_4$.
