# Supplementary material for: The external PASTA domain of the essential serine/threonine protein kinase PknB regulates mycobacterial growth
Source: Open Biol. 2015 Jul 1;5(7):150025. doi: 10.1098/rsob.150025 (PMC4632501; doi:10.1098/rsob.150025)
Supplement: Turapov 2015 supplemental material tables revised [file rsob150025supp2.pdf]

Table S1. Plasmids and strains generated for over-expression studies

Plasmid Name	Strain Name	Insert Size (bp)	Primers	Description
pMind-*pknB1*	PknB1	1881	PknBF1 & PknBR1	Full length gene
pMind *pknB5*	PknB5	1062	PknBF1 & PknBR5-TM	ΔPASTA 1-4
pMind *pknB7*	TMP	888	PknBF2 & PknBR1	TM-PASTA1-4
pMind *pknB9*	PknB9	72	PknBF2 & PknBR5-TM	TM only
pMind *pknB10*	PknB10	276	PknBF2 & PknBR4-P	TM-PASTA1
pMind *pknB11*	PknB11	480	PknBF2 & PknBR3-2P	TM-PASTA1-2
pMind *pknB12*	PknB12	684	PknBF2 & PknBR3-3P	TM-PASTA1-3
pMind *pknB13*	PknB13	819	PknBF3 & PknBR1	PASTA 1-4
pMind *pknB14*	PknB14	879	PknBF3 &MycHisR	PASTA 1-4-MycHis-tag
pMind *pknB15*	TMPH	951	PknBF1 & PknBHR2	TM-PASTA1-4- Myc-His-tag
pMind	MIND	N/A	N/A	Empty plasmid control

Table S2. Primers used in the study

N	Primer	Sequence 5'-3'	Description
1.	PknBF1	GAT*GGATCC*ATGACCACCCCTTCCCACCTGTCC	Cloning of *pknB* in pMind plasmid
2.	PknBF2	GAC*GGATCC*ATGCGTTGGGTTGCGGTGGTC	Cloning of *pknB8* in pMind plasmid
3.	PknBF3	GCA*GGATCC*ATGGGC GGCATCACCCGCGACGTTCAA	Amplification pknB10
4.	PknBR1	CGG*ACTAGT*CTACTG GCC GAA CCT CAG CGT GAT	Cloning of *pknB* in pMind plasmid
5.	PknBR4-P	TTG*ACTAGT*CTATCC GGT GGA CAC GTT GAC TGT	Cloning of *pknB* in pMind plasmid
6.	PknBR5-TM	GGT*ACTAGT*CTAGCC GAA CGT GTT GAT GGC GAT	Cloning of *pknB* in pMind plasmid
7.	PknBHR2	CGG*ACTAGT*CTA *ACG CGT* CTG GCC GAA CCT CAG CGT	Cloning of *pknB* in pMind plasmid
8.	Myc-HisF	GCA *ACG CGT* GAA CAA AAA CTC ATC TCA	Amplification of 6XHis-Myc tag
9.	Myc-HisR	GCG *ACT AGT* TAA TCT GTA TCA GGC GAA	Amplification of 6XHis-Myc tag
10.	MindF2	TGAGTCATAGTTGCACTTTATCAT	Sequencing of pMind constructs
11.	MindR3	TCCGAATCAATACGGTCGAGA	Sequencing of pMind constructs
12.	PknBR2-3P	CGG*ACTAGT* CTA CTCTTGGACACCTGTAGTTC	Cloning of *pknB12* in in pMind plasmid
13.	PknBR3-2P	CGG*ACTAGT*CTA GCCAACGATGATGAT	Cloning of *pknB11* in in pMind plasmid
14.	RT-PknBF1	TCAGAACGGAATCATCCACCGTGA	qRT-PCR
15.	RT-PknBR1	GCGATGCCGAAATCCATCACCTTT	qRT-PCR
16.	RT-PknBF2	AGAACCTCAACGTCTACGGCTTCA	qRT-PCR
17.	RT-PknBR2	ATGACGAATTGGTTGCCCTTGGAC	qRT-PCR

Table S3.Growth of *M. smegmatis* strains over-expressing *pkn*B variants

Sauton's medium				Lysogeny broth			Middlebrook 7H9 medium		
Strain	Maximum growth rate (h^{-1})	Lag-phase (h)	OD_{600nm}	Maximum growth rate (h^{-1})	Lag-phase (h)	OD_{600nm}	Maximum growth rate (h^{-1})	Lag-phase (h)	OD_{600nm}
Mind	0.12±0.010	22±2.2	1.46±0.10	0.28±0.015	21±2.2	1.14±0.15	0.12±0.010	16±1.7	0.75±0.12
PknB1	0.11±0.010	52±4.0	1.10±0.12	0.09±0.002	55±4.4	0.98±0.16	0.06±0.005	32±2.7	0.54±0.15
PknB5	0.12±0.010	46±4.0	1.50±0.15	0.11±0.008	47±4.2	1.04±0.18	0.09±0.020	21±2.0	0.61±0.15
TMP	0.11±0.010	50±2.0	1.60±0.16	0.24±0.020	29±2.0	1.05±0.10	0.10±0.030	17±1.5	0.73±0.11

Experiments were done in the Bioscreen Growth Analyser as described above. Presented data are mean ± SD from three independent experiments. The apparent lag phase was calculated as a time period from the inoculation of the culture until the OD (600 nm) was 0.1.

Table S4. Muropeptides and sugars tested in TMP growth assays.

Substance	Source	Concentration	Effect
D-glucose	Sigma	Up to 5 mM	No effect
N-acetylglucosamine (GlcNAc)	Sigma	Up to 5 mM	No effect
N-acetylmuramic acid (MurNAc)	Sigma	Up to 5 mM	No effect
GlcNAc-MurNAc disaccharide	Sigma	Up to 100 µM	No effect
MurNAc--dipeptide	Sigma	Up to 100 µM	No effect
MurNAc-pentapeptide	Synthesised in laboratory	Up to 75 µM	No effect
GlcNAc-MurNAc--pentapeptide	Synthesised in laboratory	Up to 75 µM	No effect
GlcNAc-1,6 anhydroMurNAc-pentapeptide	Synthesised in laboratory	Up to 75 µM	No effect
Tetrasaccharide- pentapeptide	Synthesised in laboratory	Up to 75 µM	No effect
Sonicated *Mtb* PG	Isolated in laboratory	Up to 1 mg/ml	20% reduction of lag-phase at 0.5 mg/ml
E. coli PG	Isolated in laboratory	Up to 1 mg/ml	20% reduction of lag-phase at 0.5 mg/ml
Lysozyme-digested *E. coli* PG	Isolated in laboratory	Up to 1 mg/ml	20% reduction of lag-phase at 0.5 mg/ml
Mutanolysin-digested *E. coli* PG	Isolated in laboratory	Up to 1 mg/ml	20% reduction of lag-phase at 0.5 mg/ml
MltA-digested *E. coli* PG	Isolated in laboratory	Up to 0.2 mg/ml	No effect
RpfB-digested *E. coli* PG	Isolated in laboratory	Up to 1 mg/ml	20% reduction of lag-phase at 0.5 mg/ml
Sonicated *M. smegmatis* PG	Isolated in laboratory	Up to 1 mg/ml	20% reduction of lag-phase at 0.5 mg/ml
RpfB-digested *M. smegmatis* PG	Isolated in laboratory	Up to 1 mg/ml	20% reduction of lag-phase at 0.5 mg/ml
Culture supernatant (Sauton's medium)	Prepared in laboratory	Up to 5xfold concentrated	Complete elimination of inhibition
Culture supernatant (7H9 supplemented medium)	Prepared in laboratory	Up to 5xfold concentrated	Complete elimination of inhibition

PG – peptidoglycan
